# A Beautiful Slide

**Published:** 1885-02

**Authors:** 


### A Beautiful Slide.

A very beautiful polariscope slide may be made, says the *Microscope*, as follows: Heat a slide until it will melt a small portion of a menthol pencil as it is drawn evenly back and forth over a perfectly clean surface. Do not use more heat than necessary to melt the material evenly. Then, as it commences to crystallize, arrest its progress frequently by passing the slide quickly over the flame of your spirit lamp; soon the cystallization will be completed, a little at a time, and a very desirable slide will be the result.

